# VEGF knockdown enhances radiosensitivity of nasopharyngeal carcinoma by inhibiting autophagy through the activation of mTOR pathway

**DOI:** 10.1038/s41598-020-73310-x

**Published:** 2020-10-01

**Authors:** Li Chen, Guoxiang Lin, Kaihua Chen, Fangzhu Wan, Renba Liang, Yongchu Sun, Xishan Chen, Xiaodong Zhu

**Affiliations:** 1 Department of Radiation Oncology, Guangxi Medical University Cancer Hospital, Nanning, 530021 Guangxi People’s Republic of China; 2 Department of Oncology, Wuming Hospital of Guangxi Medical University, Nanning, 530010 Guangxi People’s Republic of China; 3 Guangxi Key Laboratory of Early Prevention and Treatment for Regional High Frequency Tumor, Guangxi Medical University, Nanning, 530021 Guangxi People’s Republic of China

**Keywords:** Cancer, Molecular biology, Oncology

## Abstract

Vascular endothelial growth factor (VEGF) is an important pro-angiogenic factor. VEGF was reported to promote the occurrence of autophagy, which enhanced the radioresistance of tumors. The purpose of this study was to investigate the influence of VEGF silencing on the radiosensitivity of nasopharyngeal carcinoma (NPC) cells and the underlying mechanisms. The radiosensitivity of NPC cells after VEGF silencing was detected by cell counting kit 8 (CCK-8) and clonogenic assay, while cell cycle and apoptosis were detected by flow cytometry. The processes of DNA damage, repair and autophagy were examined by immunofluorescence and western blotting. The interaction between VEGF and mTOR was confirmed by western blotting and co-immunoprecipitation studies. The effect of VEGF on radiosensitivity of NPC cells was investigated in vivo using a xenograft model. Furthermore, immunohistochemistry and TUNEL assays were used to verify the relationship between autophagy and radiosensitivity in NPC after VEGF depletion. Downregulation of VEGF significantly inhibited cell proliferation and induced apoptosis of NPC cells after radiotherapy in vitro and in vivo. In addition, VEGF knockdown not only decreased autophagy level, but also delayed the DNA damage repair in NPC cells after irradiation. Mechanistically, silencing VEGF suppressed autophagy through activation of the mTOR pathway. VEGF depletion increased radiosensitivity of NPC cells by suppressing autophagy via activation of the mTOR pathway.

## Introduction

Nasopharyngeal carcinoma (NPC) is a common tumor in Southeast Asia especially in southern China, with an annual incidence of about 20 cases per 100,000 people^1^. NPC is characterized by poor tumor differentiation, complex anatomical location, and rapid invasion of adjacent organs. About 20% of patients with NPC develop local recurrence and distant metastasis after standard therapy due to radioresistance^2^. Therefore, exploring the factors that cause radioresistance of NPC and the underlying molecular mechanisms is important for improving the treatment efficacy and prognosis of NPC patients.

Vascular endothelial growth factor (VEGF) is an important pro-angiogenic factor that promotes tumor angiogenesis. VEGF causes tumor cell reoxygenation, which leads to excessive DNA replication, increased synthesis of radioresistance genes, and radioresistance^3^. Hu et al.^4^ showed that radioresistance in esophageal cancer was associated with radiation-induced abnormal secretion of VEGF, which protected tumor vessels from radiation-related damage and subsequently increased the radioresistance of tumors.

Autophagy is a conserved and essential process in organisms^5^, by which unwanted cellular contents are cleared by lysosomal degradation to maintain cell survival during stress responses^6^. Its function of scavenging intracellular waste is also one of the important mechanisms by which it inhibits tumor growth^7^. Autophagy is thought to contribute to tumor radioresistance^8,9^. Our previous study demonstrated that inhibition of autophagy enhances radiosensitivity of NPC cells^10^, which was also corroborated by Chu et al.^11^. In addition, VEGF inhibited autophagy, as evidenced by the finding that the autophagic flux and cell activity decreased significantly in VEGF-silenced tumor cells^12^.

Mammalian target of rapamycin (mTOR) is a receptor for amino acids, ATP and hormones^13^. Increasing evidence have demonstrated that mTOR inhibition activates the occurrence of autophagy^14^. Furthermore, studies have indicated that VEGF is involved in the regulation of the mTOR pathway^15,16^. However, whether VEGF regulates autophagy and affects radiosensitivity of NPC through the mTOR signaling pathway has not yet been clarified.

We have constructed the NPC radioresistant cell line CNE-2R in vitro by fractional induction of radiation on CNE-2 cells^17^; and found that inhibition of autophagy enhanced radiosensitivity of CNE-2R cells^18^. In our study, we assessed the biological function of VEGF on radiosensitivity of NPC cell lines (CNE-2R and 5-8F). In vitro and in vivo assays indicated that VEGF knockdown inhibited cell proliferation, facilitated cell apoptosis and enhanced the radiosensitivity of NPC cells. Moreover, downregulation of VEGF repressed autophagy through activation of the mTOR pathway. This study revealed the molecular regulatory mechanism of VEGF which could be a prognostic biomarker in NPC.

## Results

### VEGF expression is associated with radiosensitivity in nasopharyngeal carcinoma cells

**Figure 1:**
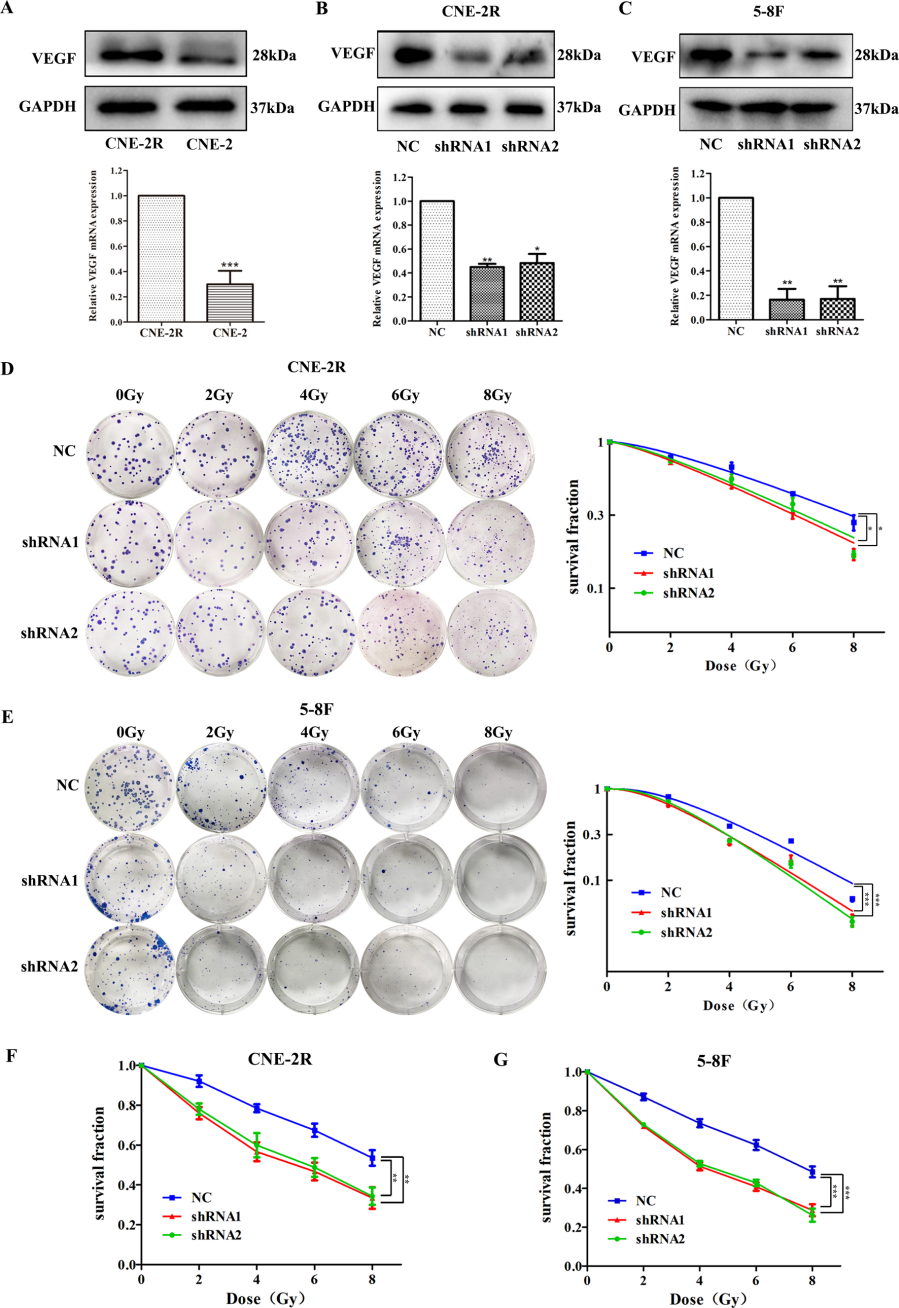
Effect of VEGF on radiosensitivity of nasopharyngeal carcinoma cells. (**A**) The expression of VEGF protein and mRNA in CNE-2R and CNE-2 cells. (**B**) The expression of VEGF protein and mRNA in CNE-2R cells treated with control or VEGF short hairpin RNAs (shRNAs). (**C**) The expression of VEGF protein and mRNA in 5-8F cells treated with control or VEGF shRNAs. (**D**, **E**) The number and size of colonies were analyzed by clonogenic assay after irradiation of NPC cells transfected with shRNAs. (**F**, **G**) Cell proliferation was detected by CCK-8 assay after irradiation of NPC cells transfected with shRNAs. *p < 0.05; **p < 0.01 and ***p < 0.001.

Our team has established the NPC radioresistant cell line CNE-2R derived from the CNE-2 cells. VEGF expression was significantly higher in CNE-2R cells than in CNE-2 cells (Fig. 1A). Therefore, we hypothesized that VEGF is necessary for radioresistance in NPC cells. To test this conjecture, we silenced VEGF with two different shRNAs, (sequences: 5′-GCGCAGCTACTGCCATCCAAT-3′ and 5′-CACAACAAATGTGAATGCAGA-3′). The negative control scramble sequence was TTCTCCGAACGTGTCACGT. As shown in Fig. 1B, C, the PCR and western blot results showed that VEGF was successfully silenced in NPC cell lines (CNE-2R and 5-8F).

To detect the effect of VEGF on radioresistance, clonogenic and CCK-8 assays were performed. The clonogenic assay (Fig. 1D, E) revealed VEGF knockdown inhibited cell survival fractions and proliferation under irradiation. The radiobiological parameters are shown in Tables 1and 2. These same results were verified using CCK-8 assay (Fig. 1F, G), which also showed that VEGF significantly increased the radioresistance of NPC cells.

**Table 1:** Radiobiological parameters of the three groups (mean ± SD).

Cell lines (CNE-2R)	D_0_	Dq	SF_2_
NC	5.091 ± 0.178	2.362 ± 0.309	0.787 ± 0.008
ShRNA1	4.31 ± 0.391	1.667 ± 0.026	0.724 ± 0.008
ShRNA2	4.193 ± 0.189	1.465 ± 0.083	0.735 ± 0.008
*p*	0.082	0.034	0.009

D_0_ is the average lethal dose, which is the dose required to hit each cell once. Dq is a quasi-threshold dose, which refers to the ability to repair sublethal injury. SF_2_ is the survival fraction at 2 Gy radiation dose. *P* < 0.05 is supposed to indicate statistical significance.

**Table 2:** Radiobiological parameters of the three groups (mean ± SD).

Cell lines (5-8F)	D_0_	Dq	SF_2_
NC	3.45 ± 0.255	2.481 ± 0.195	0.819 ± 0.031
ShRNA1	2.71 ± 0.099	1.757 ± 0.347	0.688 ± 0.051
ShRNA2	2.696 ± 0.073	2.020 ± 0.221	0.722 ± 0.037
*p*	0.002	0.039	0.018

D_0_ is the average lethal dose, which is the dose required to hit each cell once. Dq is a quasi-threshold dose, which refers to the ability to repair sublethal injury. SF_2_ is the survival fraction at 2 Gy radiation dose. *P* < 0.05 is supposed to indicate statistical significance.

### VEGF inhibits radiation-induced G2/M cell cycle arrest, apoptosis and DNA breaks

**Figure 2:**
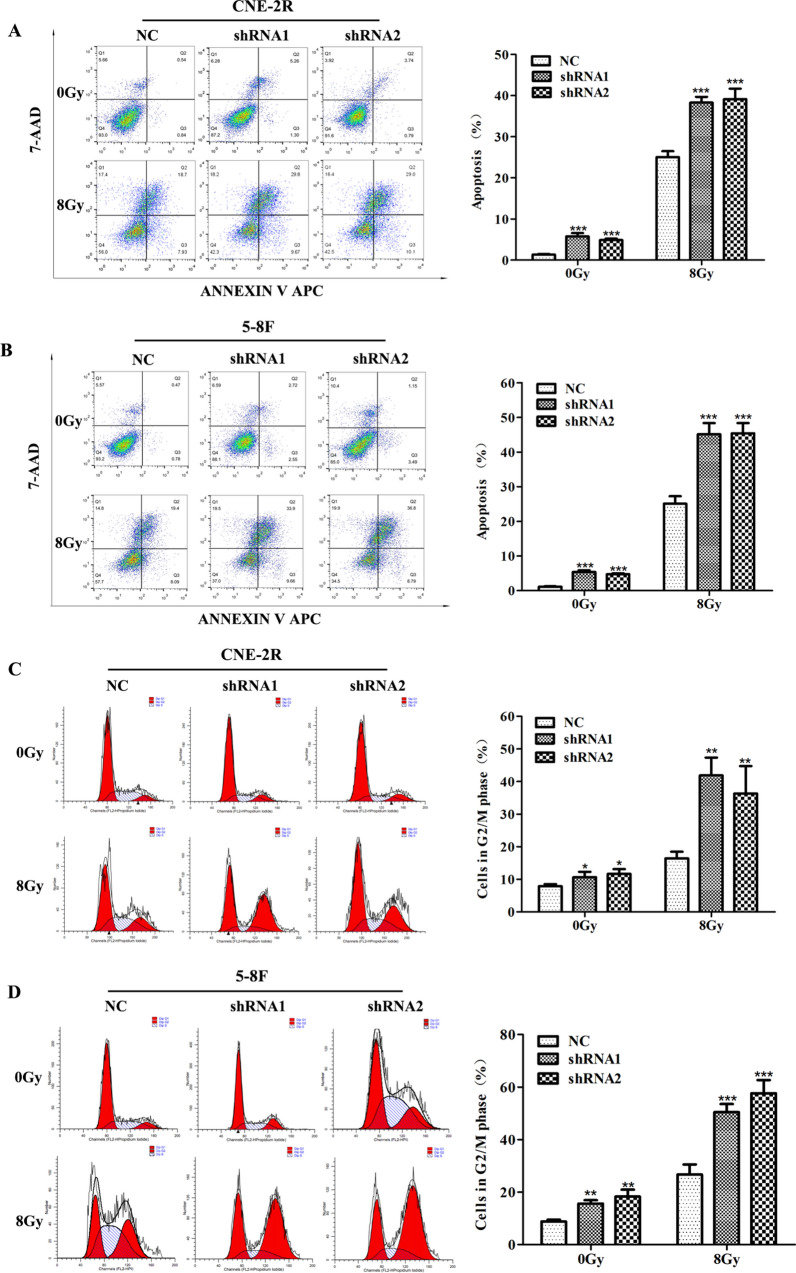
VEGF knockdown increased radiation-induced apoptosis and G2/M cell cycle arrest. (**A**, **B**) Flow cytometry was used to detect apoptosis of NPC cells with or without 8 Gy irradiation after transfection of VEGF shRNAs. (**C**, **D**) The G2/M cell cycle was evaluated by flow cytometry with or without 8 Gy irradiation of NPC cells after transfection of VEGF shRNAs. *p < 0.05; **p < 0.01 and ***p < 0.001.

To further investigate the mechanism by which VEGF inhibits cell growth, we first used flow cytometry to assess whether VEGF affects cell cycle progression and apoptosis. Annexin V-APC/7-AAD kit was used to detect the induction of apoptosis. Irrespective of 0 Gy or 8 Gy irradiation, VEGF silencing increased the rate of cell apoptosis (Fig. 2A, B), and the apoptotic cells were remarkably higher in the shRNA groups compared with the NC group. Compared with the NC group, 0 Gy irradiation in the shRNA groups caused a slight accumulation of cells in the G2/M phase, while 8 Gy irradiation in the shRNA groups remarkably increased the proportion of G2/M phase (Fig. 2C, D). This result demonstrated that VEGF silencing can induce cell cycle arrest.

**Figure 3:**
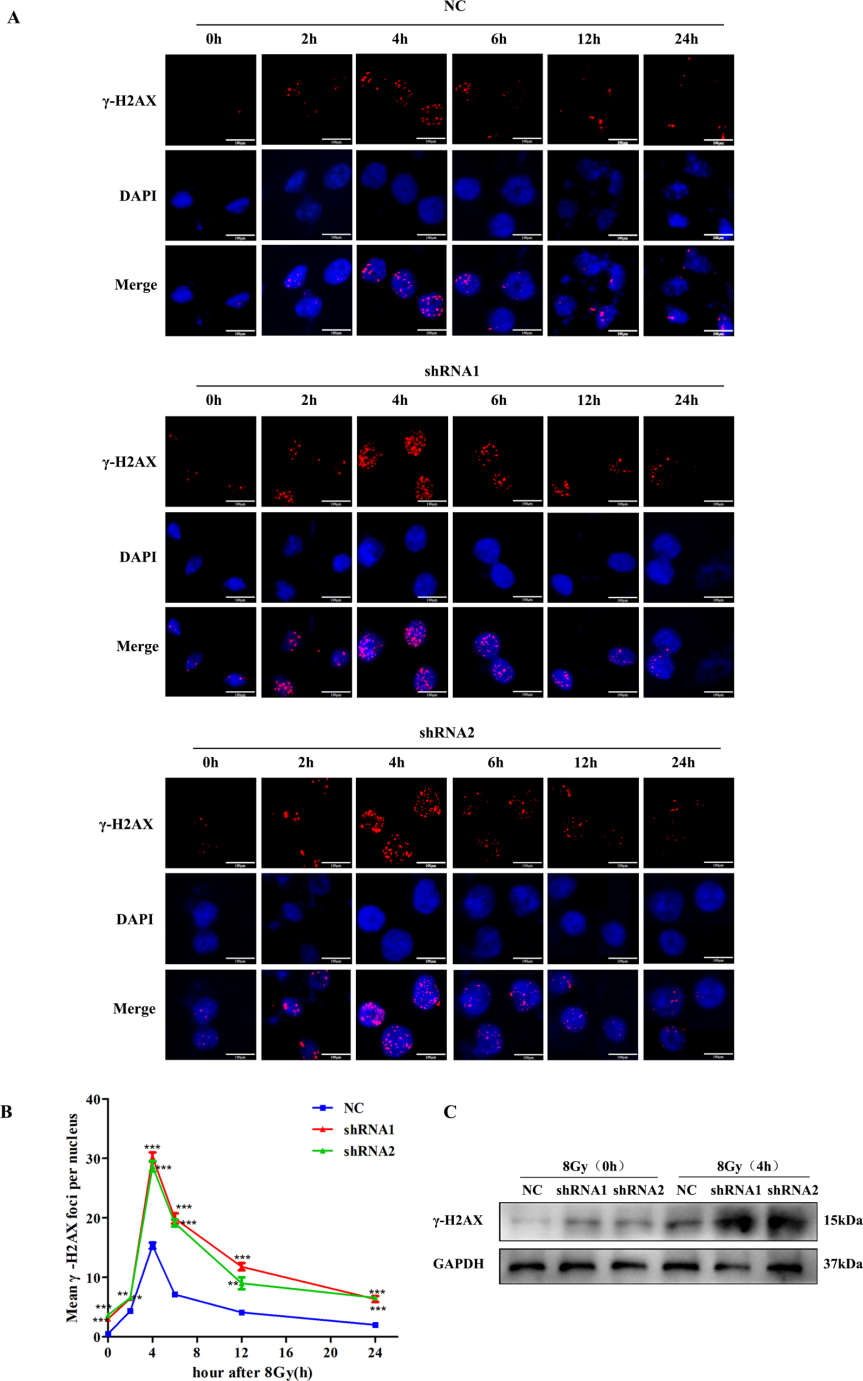
VEGF knockdown increased DNA damage in NPC cells. (**A**) Immunofluorescence for γ-H2AX at different time points after 8 Gy irradiation of CNE-2R cells after VEGF knockdown. (**B**) The statistical result of mean γ-H2AX foci per nucleus in CNE-2R cell after VEGF knockdown. (**C**) The expression of γ-H2AX protein in CNE-2R cells at oh and 4 h after 8 Gy irradiation. *p < 0.05; **p < 0.01 and ***p < 0.001, size bars = 100 μm.

To detect the influence of VEGF on DNA repair and damage, we quantified γ-H2AX foci formation by immunofluorescence staining in CNE-2R cells after VEGF knockdown at different time points (0, 2, 4, 6, 12, 24 h) after 8 Gy irradiation. As expected, the maximal foci appeared in NC and shRNA groups at 4 h after 8 Gy irradiation, which was gradually decreased to the basal level at 24 h after 8 Gy irradiation. In addition, inhibition of VEGF induced a remarkable increase in residual DNA damage, which persisted at 24 h (Fig. 3A, B). These results indicated that the repair of DNA damage was repressed in VEGF-silenced cells, which was manifested by the persistence of γ-H2AX foci from 4 to 24 h after irradiation. Western blotting analysis showed that the protein level of γ-H2AX at time point of 4 h was higher than that at 0 h, and the γ-H2AX expression in VEGF-silenced cells was higher than that in NC group (Fig. 3C), which further confirmed that VEGF depletion promoted DNA damage after irradiation. Furthermore, we also detected DNA repair pathway proteins after VEGF silencing. The expression of NHEJ DNA repair pathway proteins (ku70 and ku80) and HR DNA repair pathway proteins (BRCA1 and BRCA2) were found to be reduced in VEGF-silenced cells (Supplementary Fig. S1), confirming that inhibition of VEGF can suppress DNA repair process.

### Silencing VEGF inhibits irradiation-induced autophagy

**Figure 4:**
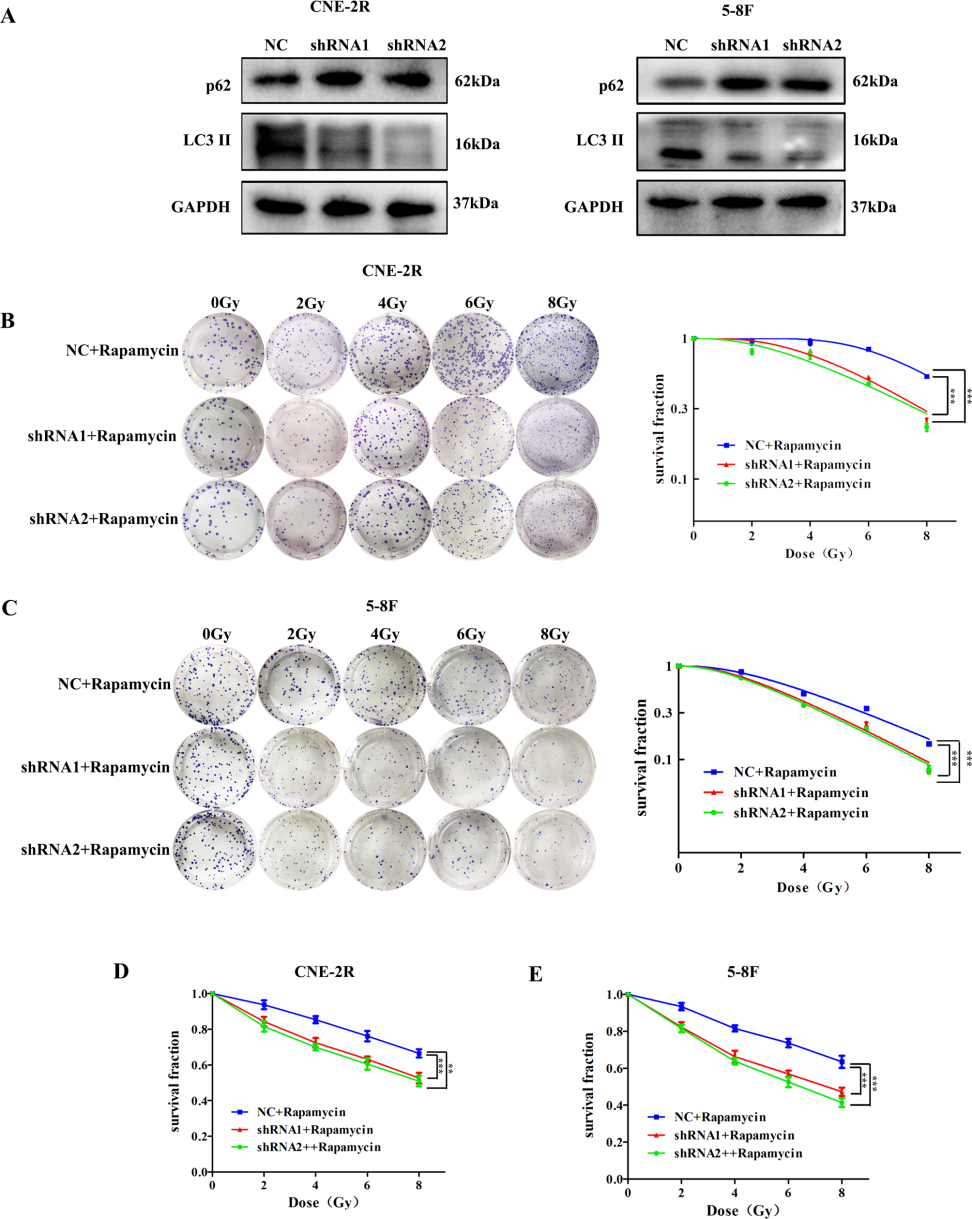
The activation of autophagy reversed the radiosensitivity induced by silencing VEGF. (**A**) The proteins of autophagy were detected by western blotting after VEGF knockdown. (**B**) The number and size of colonies were analyzed by clonogenic assay after irradiation in CNE-2R cells treated with Rapamycin. (**C**) The number and size of colonies were analyzed by clonogenic assay after irradiation in 5-8F cells treated with Rapamycin. (**D**, **E**) Cell proliferation were detected by CCK-8 assay after irradiation of NPC cells treated with Rapamycin. *p < 0.05; **p < 0.01 and ***p < 0.001.

First, we employed western blot to investigate the effect of VEGF on autophagy-related proteins without irradiation. The expression of p62 was decreased and LC3 II was increased in shRNA cells compared with NC cells (Fig. 4A). The result showed that VEGF silencing inhibited the autophagy-related proteins in NPC cells. Next, we explored whether silencing VEGF can enhance radiosensitivity of NPC cells by inhibiting autophagy. NPC cells were treated with Rapamycin, an autophagy activator, to verify whether the activation of autophagy can reverse the radiosensitivity induced by silencing VEGF through clonogenic and CCK-8 assays. Compared with the untreated cells, survival fractions and proliferation of NPC cells treated with rapamycin were enhanced (Fig. 4B–E, Supplementary Table S1 and S2). We also treated NPC cells with MHY1485 and siATG5 (sequences: 5′-GACGUUGGUAACUGACAAATTUUUGUCAGUUACCAACGUCTT-3′) to inhibit autophagy, clonogenic and CCK-8 assays showed that survival fractions and proliferation were both obviously inhibited by treatment with MHY1485 and siATG5 compared with the untreated cells (Supplementary Fig. S2 and S3, Supplementary Table S3–S6) . This result demonstrated that inhibition of autophagy increased the radiosensitivity in NPC cells after VEGF knockdown, and activation of autophagy can reverse the radiosensitivity induced by silencing VEGF. Moreover, western blotting analysis also indicated that activation of autophagy in NPC cells reversed the decrease in LC3 II and the increase in p62 caused by silencing VEGF, and inhibition of autophagy had the opposite effect (Supplementary Fig. S4).

**Figure 5:**
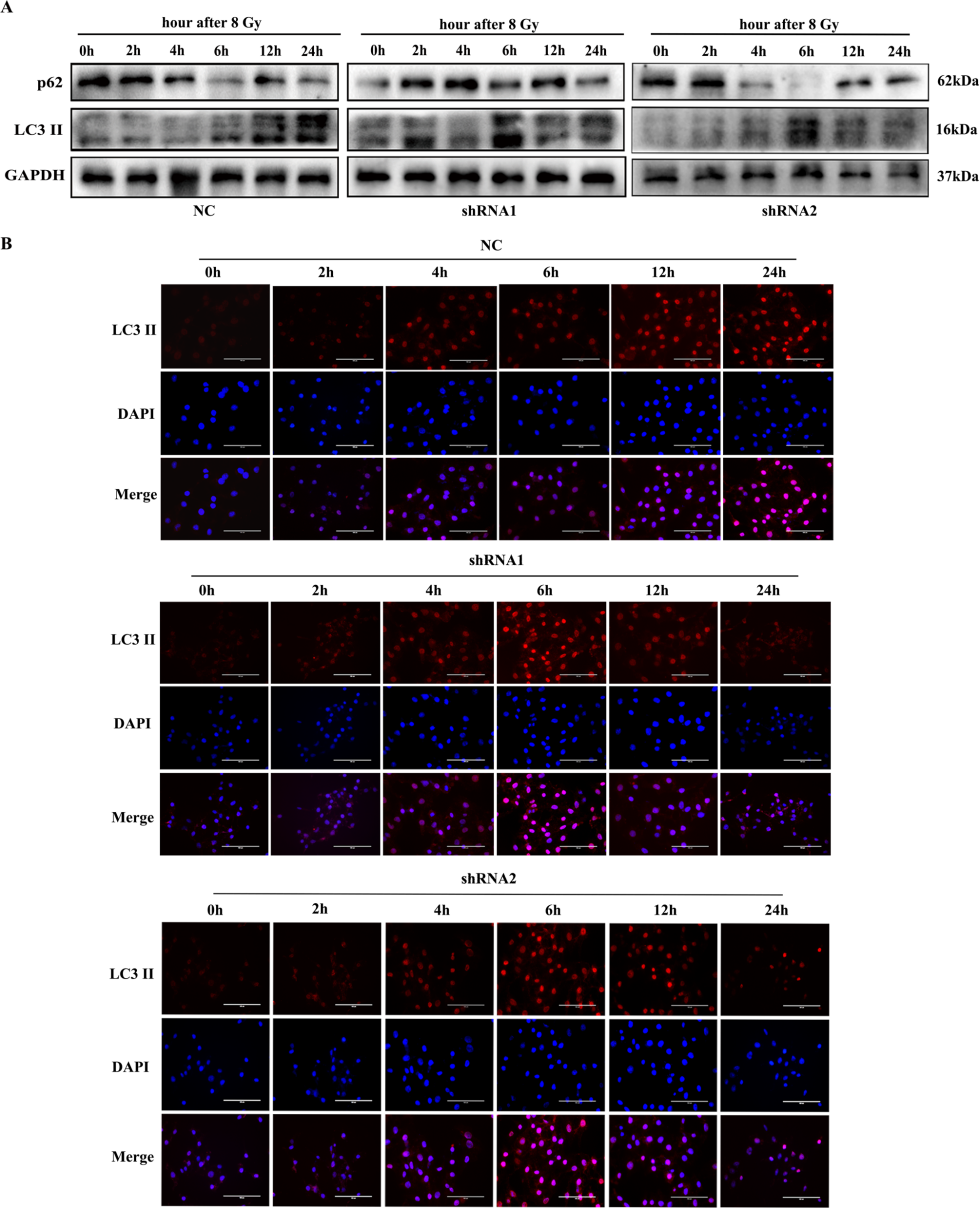
VEGF knockdown inhibited radiation-induced autophagy. (**A**) The proteins of autophagy in CNE-2R cells at different time points after 8 Gy irradiation. (**B**) Effect of VEGF on autophagy detected by immunofluorescence of LC3 II at different time points after 8 Gy irradiation. Size bars = 100 μm.

To investigate whether VEGF influenced the activity of autophagy induced by irradiation, western blot and immunofluorescence staining were utilized to observe autophagic activity in CNE-2R cells after VEGF knockdown at different time points (0, 2, 4, 6, 12, 24 h) after 8 Gy irradiation. As shown in Fig. 5A, the western blot result indicated that LC3 II increased gradually with time and peaked at 24 h after 8 Gy irradiation in the NC group, while p62 declined gradually over time. However, in the shRNA groups, the expression level of LC3 II peaked at 6 h, while p62 was relatively low at 6 h after 8 Gy irradiation. Moreover, immunofluorescence assay was employed to detect the formation of LC3 II puncta in CNE-2R cells. After 8 Gy irradiation, the number of LC3 II puncta increased significantly in the NC cells at 24 h, and in the shRNA cells at 6 h (Fig. 5B). Overall, these results showed that VEGF knockdown inhibited radiation-induced autophagy in NPC cells.

### VEGF regulates autophagy through the mTOR pathway

**Figure 6:**
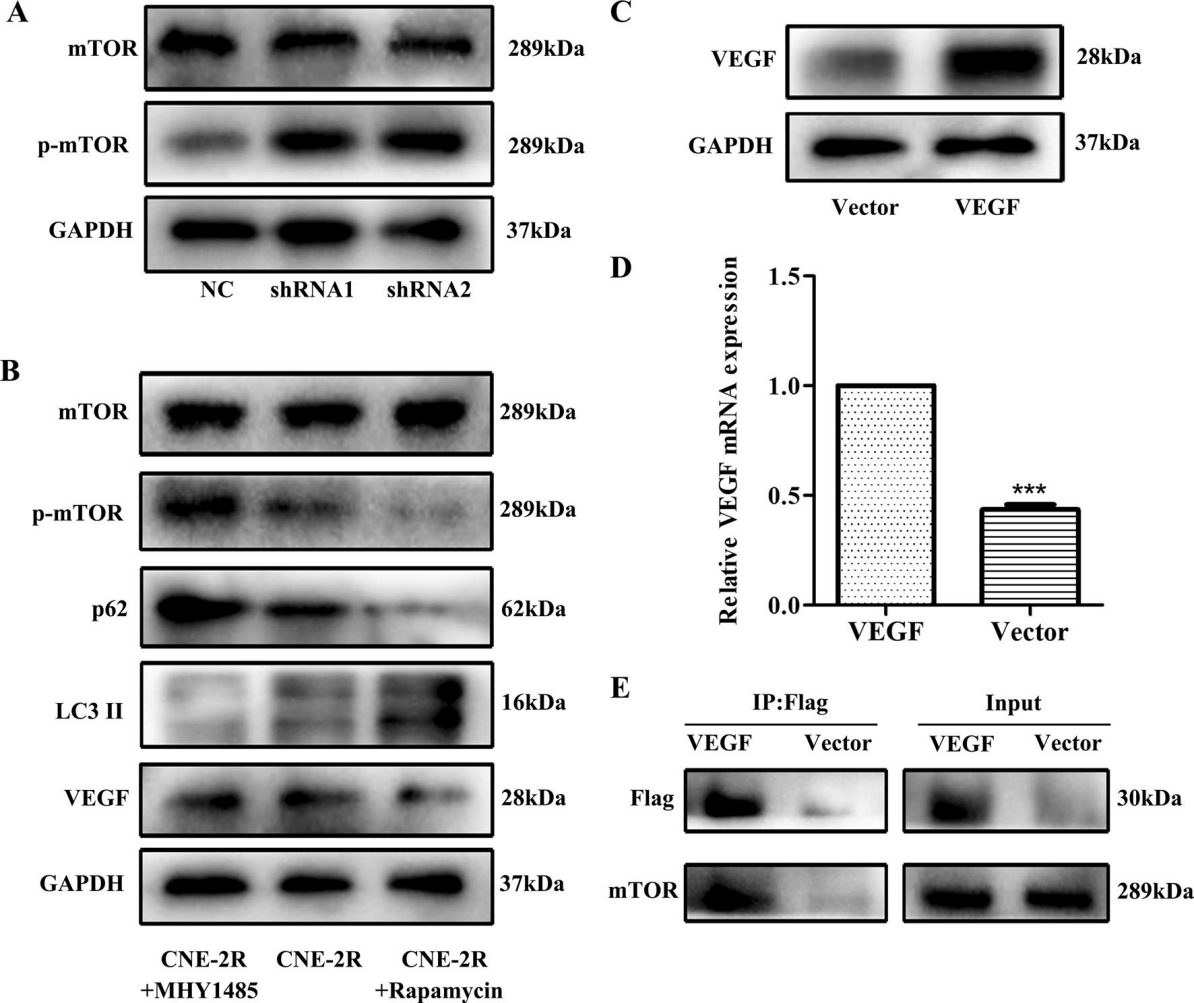
VEGF regulated autophagy through the mTOR pathway. (**A**) The expression of mTOR and p-mTOR proteins in CNE-2R cells after VEGF knockdown. (**B**) The protein levels of mTOR, p-mTOR, LC3II, p62 and VEGF were detected by western blotting after treatment of CNE-2R cells with mTOR activator (MHY1485) and inhibitor (Rapamycin). (**C**, **D**) VEGF over-expression vector was transfected into CNE-2R cells. Transfection efficiencies were tested by western blotting and qPCR assays. (**E**) The formation of the VEGF/mTOR complex was detected by Co-IP.

mTOR is known to be related to autophagy, so we further explored whether VEGF regulates autophagy through the mTOR pathway in NPC cells. First, western blotting confirmed that phosphorylated mTOR declined significantly after silencing VEGF, whereas total mTOR protein remained almost unchanged (Fig. 6A). In order to further verify the correlation between VEGF and mTOR, VEGF was over-expressed in CNE-2R cells (Fig. 6C, D). Co-IP assay confirmed that VEGF co-precipitated with mTOR (Fig. 6E), suggesting that VEGF interacted with mTOR. Autophagy was suppressed by the mTOR activator (MHY1485) and enhanced by the mTOR inhibitor (Rapamycin) in CNE-2R cells (Fig. 6B). The above findings demonstrated that VEGF knockdown inhibited autophagy by activating the mTOR pathway in NPC cells.

### VEGF can regulate autophagy to enhance radioresistance in vivo

**Figure 7:**
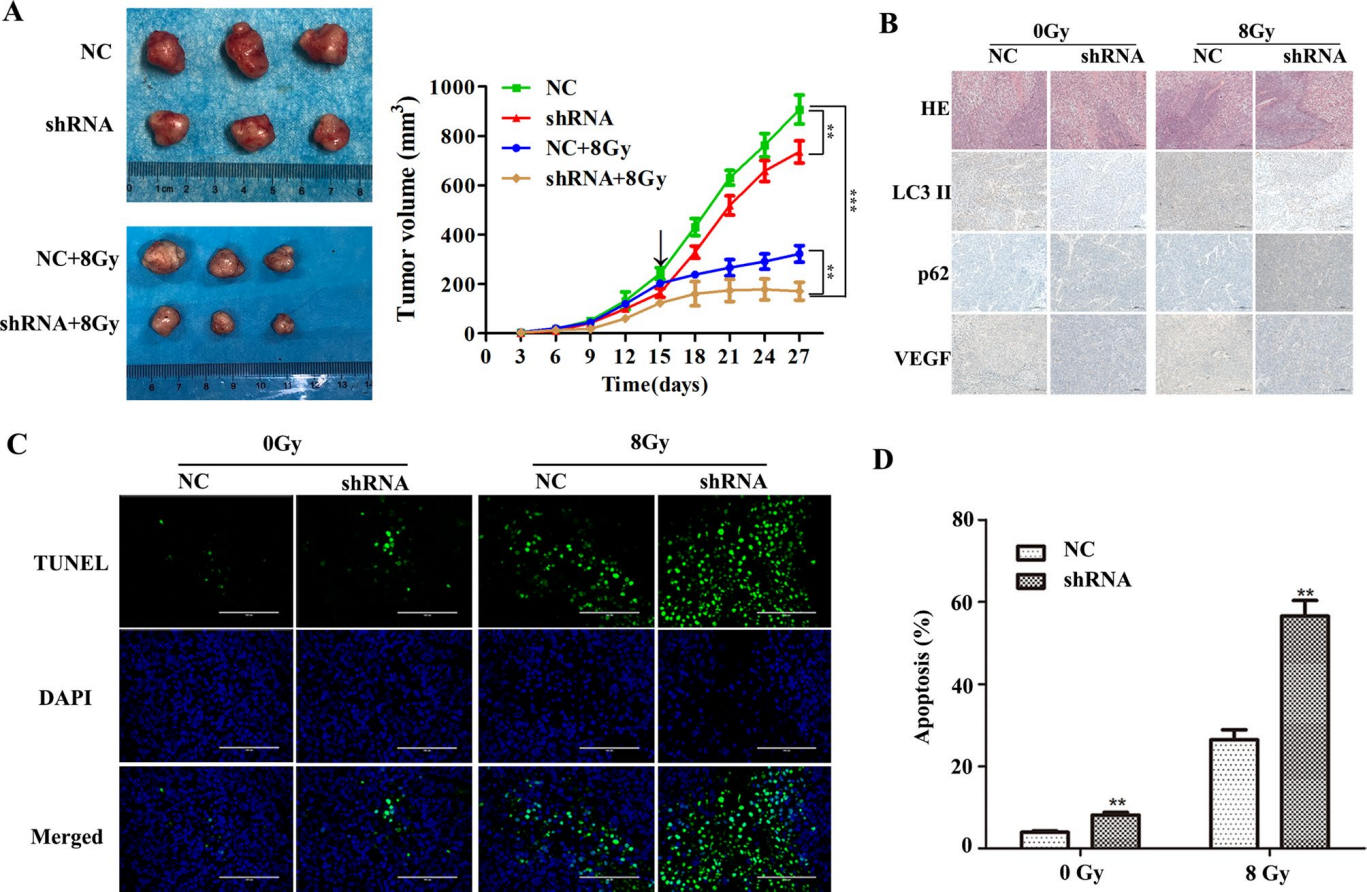
VEGF knockdown promoted radiosensitivity in nude mouse model. (**A**) Tumor size was measured at 27 days after subcutaneous inoculation of NC and shRNA cells in nude mice, and the growth curve of tumor nodules with or without 8 Gy irradiation in each group (black arrow indicates the time point of radiation). (**B**) Expression of LC3 II, p62 and VEGF was analyzed by IHC assay. (**C**) Tumor nodules were examined by TUNEL assay. (**D**) Quantitative analysis of TUNEL assay results. **p < 0.01 and ***p < 0.001, size bars = 100 μm.

Our in vitro studies verified that VEGF can affect radiosensitivity of nasopharyngeal carcinoma by regulating autophagy. To further investigate these effects in vivo, CNE-2R cells transfected with shRNAs were injected into nude mice. Half of the nude mice in shRNA group and the NC group were randomly selected for 8 Gy irradiation on day 15. We found that inhibition of VEGF marginally repressed tumor growth, and VEGF-silenced tumors displayed dramatically diminished tumor volume with irradiation, further supporting that the inhibiting function of VEGF silencing on tumor growth was significantly enhanced under irradiation (Fig. 7A). Irrespective of 0 Gy or 8 Gy irradiation, the IHC assays confirmed that LC3 II and VEGF expression levels were much higher in the NC group than in the shRNA group, while p62 expression showed the opposite trend (Fig. 7B). The percentages of apoptotic cells were tested by TUNEL staining in tumor sections (Fig. 7C, D). Compared with the NC cells, the apoptosis level of the shRNA cells increased slightly after 0 Gy irradiation, but significantly increased after 8 Gy irradiation. These results showed that silencing VEGF enhanced the irradiation sensitivity of NPC cells in vivo, which was in accordance with its functions in vitro.

## Discussion

At present, radiotherapy is one of the main treatments for NPC. However, during the treatment course, treatment failure often occurs due to radiation resistance. Therefore, it is particularly important to explore the mechanism of radioresistance in NPC patients. Autophagy is the most common intracellular degradation process in organisms, which can maintain normal homeostasis and influence tumor development. The anti-stress role of autophagy is important for cancer cell survival during therapy^19,20^. VEGF was highly expressed in the radioresistant NPC cell line CNE-2R as compared to CNE-2 cells. Therefore, this study mainly explored the possible relationships and mechanisms of VEGF and radioresistance in NPC.

Human VEGF gene is located on 6p21.3 chromosome, VEGF has disulfide bridges in its protein structure^21,22^. Normal angiogenesis is in a dynamically balanced homeostasis, but becomes abnormally active in tumor tissues. The most important driver of tumor angiogenesis is VEGF^23^. In 1993, Ferrara et al.^24^ found that inhibition of VEGF blocked tumor growth. Moreover, knocking down VEGF could significantly inhibit the growth of tumor cells^25,26^. Radioresistance is a major obstacle to cancer treatment, and is thought to be involved in multiple signaling pathways in cancer^27^. In 1999, Gorski et al. found that irradiation increased the secretion of VEGF in tumor cells, and VEGF could enhance the radioresistance of endothelial cells^28^. Similarly, Chen et al.^29^ and Kil et al.^30^ found that radiation therapy of tumors induces increased VEGF secretion. Moreover, the relationship between high VEGF expression in tumor tissues and radiation therapy has been demonstrated in many cancers^31–33^. Consistent with previous reports, our results showed that inhibition of VEGF significantly repressed the tumor growth especially with irradiation, which indicated that VEGF could promote the radioresistance of NPC cells.

Ionizing radiation-induced cell killing is mainly associated with DNA double-strand breaks (DSB) and cell cycle redistribution. Upon exposure to ionizing radiation, cells produce reactive oxygen species (ROS) and free radicals that induce DNA damage, the most severe being DSB^34^. The cancer cells die of necrosis and apoptosis after irradiation, so radiosensitivity can be measured by the degree of apoptosis^35^. Therefore, this study demonstrated that NPC cells had a higher apoptotic rate after silencing VEGF especially with irradiation using flow cytometry and TUNEL assays.

Since DSBs cause cell death after irradiation, tumor cells can induce radioresistance by activating DNA repair and cell cycle checkpoints^36^. The G2 phase of the cell cycle is a late stage of DNA synthesis and numerous proteins are required for mitosis phase^37^. The cell cycle is an important indicator of electro-radiation sensitivity and the G2/M phase cells have the highest radiosensitivity. Hence, partial simultaneous radiotherapy of cells during the radiosensitivity phase of the cell cycle is more effective^38^. Cui et al.^39^ found that arsenic trioxide enhances radiosensitivity by promoting G2/M phase arrest in esophageal cancer cells, reducing the repair of sublethal damage and inducing apoptosis. This study confirmed that NPC cells were arrested in the G2/M phase after silencing VEGF. There is a linear correlation between γ-H2AX foci and DSBs, and the formation and disappearance kinetics of γ-H2AX are consistent with the successful repair of DSBs^40^. In the present study, IF assay showed that γ-H2AX expression peaked about 4 h after irradiation, and the residual γ-H2AX was observed in VEGF-silenced cells for 24 h. NHEJ and HR are the main pathways for DNA DSB repair^41^. Hence, these pathways were we also detected in VEGF silenced cells in this study. The representative proteins Ku70 and Ku80 (NHEJ pathway), and BRCA1 and BRCA2 (HR pathway) were significantly decreased, suggesting that DNA repair pathways plays an important role in the radioresistance of NPC, and VEGF silencing can weaken DNA repair and increase DNA DSB to induce radiation sensitivity in NPC cells.

In the process of tumor development, autophagy can protect tumor cells from cancer therapy by opposing pressure, providing energy, and regulating homeostasis^42–45^. Furthermore, increasing evidences have confirmed the existence of autophagy as the basic mechanism of cell survival in stressful environments caused by radiotherapy and chemotherapy. The increase of autophagy enhances the radioresistance of tumors, and tumors with autophagy defect are more sensitive to radiotherapy^46^. The expression of autophagy-related genes light chain 3 (LC3), Beclin-1, ATG5 and p62 could be used as indicators of tumor recurrence and poor prognosis^47^. The transition from LC3 I to LC3 II is considered to be a sign of autophagy activity. P62 is another marker of autophagy, which is known as polypeptide binding protein chelator 1. The expression level of p62 increased when autophagy was inhibited, and decreased when autophagy was induced^48^. This study showed that VEGF silencing could inhibit autophagy. Since autophagy is a dynamic process, we measured autophagy-related proteins at different time points after irradiation by western blotting and IF. Interestingly, knockdown of VEGF facilitated the occurrence and disappearance of autophagy in NPC cells after irradiation, and decreased the duration of autophagy. Moreover, treatment of NPC cells with autophagy activator reversed the radiosensitivity induced by VEGF silencing. These results indicated that knockdown of VEGF can enhance radiosensitivity of NPC by suppressing autophagy.

As autophagy is regulated by an intricate network, and the mTOR pathway is activated to negatively regulate autophagy^49^. Recent studies have shown that the mTOR pathway is linked to tumor development and treatment resistance^50–52^. In this study, silencing VEGF increased mTOR phosphorylation. Similarly, Co-IP assay also confirmed the relationship between VEGF and mTOR. In addition, we used mTOR activator (MHY1485) and inhibitor (Rapamycin) to evaluate their effects on VEGF-induced autophagy in radioresistant NPC cells. As expected, MHY1485 activated the expression of p-mTOR and p62, and inhibited the expression of LC3 II in CNE-2R cells, while rapamycin showed the opposite effect. These findings indicated that silencing VEGF inhibited the autophagy-related proteins by activating the mTOR pathway.

In summary, this study showed that inhibition of VEGF exerted anti-tumor effects and increased the radiosensitivity of NPC cells. In addition, the role of VEGF in radioresistance was closely related with the induction of autophagy. Moreover, silencing VEGF inhibited autophagy by activating the mTOR pathway. Taken together, these findings demonstrated that inhibition of VEGF-mediated autophagy may provide a better therapeutic strategy for addressing radioresistance in NPC.

## Methods

### Cell lines and transfection

Cells were maintained in DMEM medium containing 10% fetal bovine serum (FBS) at 37 °C in 5% CO_2_ incubator. The GV248-Puromycin-EGFP-shRNA-VEGF lentiviral vectors and GV358-3FLAG-Puromycin-EGFP-VEGF lentiviral vectors were transfected into NPC cells. The ATG5 siRNA was transfected into NPC cells by using Lipofectamine 3000 (Invitrogen, Carlsbad, CA) . The transfection effect was verified by qPCR and western blotting.

### RT-qPCR analysis

Total RNA was isolated from cells with TRIzol reagent (Takara, Dalian, Japan), and then reverse transcribed into cDNA. The SYBR Premix Ex Taq II (Takara, Dalian, Japan), dH_2_O, cDNA, and primers were mixed to prepare a qPCR reaction system, and a RT-qPCR reaction was performed. The relative quantitative analysis of mRNA was conducted by the 2^−ΔΔct^ method. The following primer sequences were used: (5′ → 3′): VEGF-F: TCACAGGTACAGGGATGAGGACAC, VEGF-R: CAAAGCACAGCAATGTCCTGAAG; GAPDH-F:CAGGAGGCATTGCTGATGAT, GAPDH-R: GAAGGCTGGGGCTCATTT.

### Western blot analysis

Total protein was isolated from cells by RIPA protein lysate. Quantitative detection of protein was conducted by the BCA method. Thereafter, 20 μg of protein sample was added to appropriate amount of 4× loading buffer and denatured by heating. After electrophoresis by SDS-PAGE (6–12%), the proteins were then transferred to PVDF membranes and blocked with 5% skimmed milk. The corresponding primary and secondary antibodies were incubated with the membranes. The bands were visualized with enhanced chemiluminescence (ECL) and analysed with ImageJ software.

### Clonogenic assay

Cells at densities of 200, 200, 400, 600, 1000 cells/well were added to a six-well plate according to the 0, 2, 4, 6 and 8 Gy irradiation doses. After 24 h of culture, cells were subjected to different irradiation with 6 MV X-rays, and then cultured for 12 days. The medium was discarded, and the cells were fixed with 4% paraformaldehyde and stained with Giemsa stain. More than 50 cells were considered to be an effective colony, and the relevant parameters were calculated, clonal formation rate (PE) = number of effective colonies / number of cells inoculated, survival fraction (SF) = PE of irradiated cells / PE of non-irradiated cells. Based on the multi-target model, we fitted the cell survival curve by using the GraphPad Prism 5.0 software, and D_0_ (= 1/k), Dq (= lnN × D_0_) and SF_2_ were calculated.

### Cell counting kit 8

Cell suspensions at 4 × 10^3^ cells/well were added to 96-well plates. After incubating for 24 h, the cells were subjected to different irradiation with 6 MV X-rays and then cultured for another 48 h. After that, adding 10 μL of CCK-8 (Dojindo, Shanghai, China) for every 100 μL medium and incubating for 1 h. The microplate reader was used to measure the absorbance value of each group at 450 nm and the SF of cell was calculated.

### Cell cycle analysis

After all groups of cells were irradiated with 0 Gy or 8 Gy with 6 MV X-rays for 48 h, 5 × 10^5^ cells were collected from each group and washed with PBS. Each group was fixed with 1 mL of 75% pre-cooled ethanol at − 20 °C overnight, and washed with PBS. Then, 0.5 mL PI/RNA staining buffer (BD, New Jersey, USA) was added for 20 min. All cells were filtered with a nylon filter and detected by flow cytometer.

### Apoptosis analysis

After all groups of cells were irradiated with 0 Gy or 8 Gy with 6 MV X-rays for 48 h, 5 × 10^5^ cells were collected from each group and washed with PBS. After that, cell suspension were added 7 μL Annexin V-APC /7-AAD (BD, New Jersey, USA) for 20 min. Thereafter, the cells were filtered with a nylon filter and detected by flow cytometer.

### Immunofluorescence (IF)

The two groups of cell suspensions were inoculated on coverslips, and they were irradiated with 8 Gy after 24 h of culture. At indicated time points (0, 2, 4, 6, 12 and 24 h), 4% paraformaldehyde was used to fix the cells, which then treated with Immunol staining blocking buffer for blocking. Next, the NPC cells were incubated with primary and secondary antibodies. Finally, nuclei were counterstained with DAPI solution, and coverslips were mounted with Antifade polyvinylpyrrolidone mounting medium. The EVOS FL Auto microscope was used to take images, and the number of cells which expressed the target proteins in the cytoplasm and nucleus were counted.

### Co-immunoprecipitation (Co-IP)

The proteins of the vector group and the VEGF group were extracted separately, and the specific steps are as described above. 40 μL of Anti-Flag M2 Affinity Gel was thoroughly mixed with 1 mL PBS, centrifuged and discarded the supernatant. 500 μL of total proteins were mixed with the above precipitate, and then incubated them overnight at 4 °C. Subsequently, the mixture was centrifuged again and discarded the supernatant. 4 × SDS-PAGE binding buffer were put into the precipitate and denatured by heating at 100 °C. Then, the next steps of gel electrophoresis were the same as that of western blot analysis.

### In vivo nude mouse models

All of the experimental procedures involving animals were approved by the Ethics Committee of Guangxi Medical University Cancer Hospital (No. LW2020001) and were performed in strict accordance with the guidelines of the Research Animal Care Committee of Affiliated Tumor Hospital of Guangxi Medical University. Male athymic 4-week-old BALB/C nude mice were housed under specific pathogen free (SPF) conditions in Experimental Animal Center of Guangxi Medical University. Two group cells (4 × 10^6^) were implanted into the right groin of nude mice. There are 6 mice in each group, and each mouse is marked with an ear tag. When the tumor diameter was about 1 cm, 3 mice from each group were randomly selected for 8 Gy irradiation. All mice were sacrificed after observation for about 12 days after irradiation. During the periodical measurement of tumor size, calculate tumor volume according to the following formula: V = width^2^ × length × 0.5. Tumor tissues were paraffin-embedded for haematoxylin and eosin (H&E) staining.

### Immunohistochemistry (IHC)

The paraffin-embedded tissue slides were baked in 60 °C incubator for 2 h, and then dewaxed, hydrated, antigen recovered and the endogenous peroxidase was eliminated. Subsequently, the primary and secondary antibodies were droped on the slides and incubated. The staining intensity was recorded: 0 (no staining), 1 (light yellow), 2 (yellow), 3 (brownish yellow). The percentage of stained cells was recorded: 1 (0–25%), 2 (26–50%), 3 (51–75%) and 4 (76–100%). Then multiply the score of staining intensity and percentage stained cells to get the immunoreactivity score of each tissue, and it was classified into two grades: low (0–3) or high (4–7).

### TUNEL

TUNEL assay was detected by the commercial Tunel kit (Roche, Basel, Switzerland) according to the instruction of manufacture. The tunel-positive cells were regarded as apoptotic cells by observing the sections on the fluorescence microscope and analyzing five randomly selected regions of each slide. The apoptotic index was calculated as a percentage of apoptotic nuclei to total nuclei.

### Statistical analysis

All statistical results were analysed using SPSS 17.0 and GraphPad Prism 5.0 software. All data are expressed as means ± standard deviation. Statistical *p* values were analysed by Student’s *t* tests or one-way ANOVAs. *P* < 0.05 was supposed to indicate statistical significance. Each experiment was repeated three times. All methods were performed in accordance with the relevant guidelines and regulations.

## Supplementary information

Supplementary information.

## References

[1] Cao SM, Simons MJ, Qian CN (2011). The prevalence and prevention of nasopharyngeal carcinoma in China. Chin. J. Cancer.

[2] Feng XP (2010). Identification of biomarkers for predicting nasopharyngeal carcinoma response to radiotherapy by proteomics. Can. Res..

[3] Cohen-Jonathan Moyal E (2009). Angiogenic inhibitors and radiotherapy: From the concept to the clinical trial. Cancer Radiother..

[4] Hu L (2019). Apatinib enhances the radiosensitivity of the esophageal cancer cell line KYSE-150 by inducing apoptosis and cell cycle redistribution. Oncol. Lett..

[5] Yu L, Chen Y, Tooze SA (2018). Autophagy pathway: Cellular and molecular mechanisms. Autophagy.

[6] Wu Y (2018). The role of autophagy in colitis-associated colorectal cancer. Signal Transduc. Targeted Ther..

[7] Mah LY, Ryan KM (2012). Autophagy and cancer. Cold Spring Harbor Perspect. Biol..

[8] Chang L (2014). PI3K/Akt/mTOR pathway inhibitors enhance radiosensitivity in radioresistant prostate cancer cells through inducing apoptosis, reducing autophagy, suppressing NHEJ and HR repair pathways. Cell Death Dis..

[9] Yuan X (2015). Suppression of autophagy augments the radiosensitizing effects of STAT3 inhibition on human glioma cells. Exp. Cell Res..

[10] Liang ZG (2018). The role of autophagy in the radiosensitivity of the radioresistant human nasopharyngeal carcinoma cell line CNE-2R. Cancer Manag. Res..

[11] Chu C, Niu X, Ou X, Hu C (2019). LAPTM4B knockdown increases the radiosensitivity of EGFR-overexpressing radioresistant nasopharyngeal cancer cells by inhibiting autophagy. OncoTargets Ther..

[12] Stanton MJ (2013). Autophagy control by the VEGF-C/NRP-2 axis in cancer and its implication for treatment resistance. Can. Res..

[13] Lund-Ricard Y, Cormier P, Morales J, Boutet A (2020). mTOR signaling at the crossroad between metazoan regeneration and human diseases. Int. J. Mol. Sci..

[14] Al‐Bari MAA, Xu P (2020). Molecular regulation of autophagy machinery by mTOR-dependent and -independent pathways. Ann. N.Y. Acad. Sci..

[15] Yan X (2019). EG-VEGF silencing inhibits cell proliferation and promotes cell apoptosis in pancreatic carcinoma via PI3K/AKT/mTOR signaling pathway. Biomed. Pharmacother. Biomed. Pharmacother..

[16] Patel AB, Tsilioni I, Weng Z, Theoharides TC (2018). TNF stimulates IL-6, CXCL8 and VEGF secretion from human keratinocytes via activation of mTOR, inhibited by tetramethoxyluteolin. Exp. Dermatol..

[17] Guo Y (2012). Identification of genes involved in radioresistance of nasopharyngeal carcinoma by integrating gene ontology and protein-protein interaction networks. Int. J. Oncol..

[18] Zhou ZR (2013). Poly(ADP-ribose) polymerase-1 regulates the mechanism of irradiation-induced CNE-2 human nasopharyngeal carcinoma cell autophagy and inhibition of autophagy contributes to the radiation sensitization of CNE-2 cells. Oncol. Rep..

[19] Maycotte P, Thorburn A (2011). Autophagy and cancer therapy. Cancer Biol. Ther..

[20] Klionsky DJ, Emr SD (2000). Autophagy as a regulated pathway of cellular degradation. Science.

[21] Yamazaki Y, Morita T (2006). Molecular and functional diversity of vascular endothelial growth factors. Mol. Divers..

[22] Takahashi H, Shibuya M (2005). The vascular endothelial growth factor (VEGF)/VEGF receptor system and its role under physiological and pathological conditions. Clin. Sci..

[23] Hicklin DJ, Ellis LM (2005). Role of the vascular endothelial growth factor pathway in tumor growth and angiogenesis. J. Clin. Oncol..

[24] Ferrara N (2011). From the discovery of vascular endothelial growth factor to the introduction of avastin in clinical trials—An interview with Napoleone Ferrara by Domenico Ribatti. Int. J. Dev. Biol..

[25] Luo M (2016). VEGF/NRP-1axis promotes progression of breast cancer via enhancement of epithelial-mesenchymal transition and activation of NF-kappaB and beta-catenin. Cancer Lett..

[26] Jendreyko N, Popkov M, Rader C, Barbas CF (2005). Phenotypic knockout of VEGF-R2 and Tie-2 with an intradiabody reduces tumor growth and angiogenesis in vivo. Proc. Natl. Acad. Sci. USA.

[27] Yang L (2015). EBV-LMP1 targeted DNAzyme enhances radiosensitivity by inhibiting tumor angiogenesis via the JNKs/HIF-1 pathway in nasopharyngeal carcinoma. Oncotarget.

[28] Gorski DH (1999). Blockage of the vascular endothelial growth factor stress response increases the antitumor effects of ionizing radiation. Can. Res..

[29] Chen YH (2014). Radiation-induced VEGF-C expression and endothelial cell proliferation in lung cancer. Strahlentherapie und Onkologie: Organ der Deutschen Rontgengesellschaft ... [et al].

[30] Kil WJ, Tofilon PJ, Camphausen K (2012). Post-radiation increase in VEGF enhances glioma cell motility in vitro. Radiat. Oncol..

[31] Park I, Yang H, Park JS, Koh GY, Choi EK (2018). VEGF-Grab enhances the efficacy of radiation therapy by blocking VEGF-A and treatment-induced PlGF. Int. J. Radiat. Oncol. Biol. Phys..

[32] Zhang N (2016). Rationally combining anti-VEGF therapy with radiation in NF2 schwannoma. J. Rare Dis. Res. Treat..

[33] Gao X (2015). Anti-VEGF treatment improves neurological function and augments radiation response in NF2 schwannoma model. Proc. Natl. Acad. Sci. USA.

[34] Roos WP, Kaina B (2013). DNA damage-induced cell death: from specific DNA lesions to the DNA damage response and apoptosis. Cancer Lett..

[35] Rahmanian N, Hosseinimehr SJ, Khalaj A (2016). The paradox role of caspase cascade in ionizing radiation therapy. J. Biomed. Sci..

[36] Morgan MA, Lawrence TS (2015). Molecular pathways: overcoming radiation resistance by targeting DNA damage response pathways. Clin. Cancer Res..

[37] Hartwell LH, Weinert TA (1989). Checkpoints: Controls that ensure the order of cell cycle events. Science.

[38] Pawlik TM, Keyomarsi K (2004). Role of cell cycle in mediating sensitivity to radiotherapy. Int. J. Radiat. Oncol. Biol. Phys..

[39] Cui YH (2012). Radiosensitivity enhancement by arsenic trioxide in conjunction with hyperthermia in the EC-1 esophageal carcinoma cell line. Asian Pac. J. Cancer Prev..

[40] Banáth JP, MacPhail SH, Olive PL (2004). Radiation sensitivity, H2AX phosphorylation, and kinetics of repair of DNA strand breaks in irradiated cervical cancer cell lines. Can. Res..

[41] Jackson SP (2002). Sensing and repairing DNA double-strand breaks. Carcinogenesis.

[42] Hait WN, Jin S, Yang JM (2006). A matter of life or death (or both): understanding autophagy in cancer. Clin. Cancer Res..

[43] Zhou S (2012). Autophagy in tumorigenesis and cancer therapy: Dr. Jekyll or Mr. Hyde?. Cancer Lett..

[44] Mathew R (2009). Autophagy suppresses tumorigenesis through elimination of p62. Cell.

[45] Singh SS (2018). Dual role of autophagy in hallmarks of cancer. Oncogene.

[46] Wang F (2018). Chloroquine enhances the radiosensitivity of bladder cancer cells by inhibiting autophagy and activating apoptosis. Cell. Physiol. Biochem..

[47] Terabe T (2018). Expression of autophagy-related markers at the surgical margin of oral squamous cell carcinoma correlates with poor prognosis and tumor recurrence. Hum. Pathol..

[48] Pankiv S (2007). p62/SQSTM1 binds directly to Atg8/LC3 to facilitate degradation of ubiquitinated protein aggregates by autophagy. J. Biol. Chem..

[49] Zhang Y (2018). Thymoquinone inhibits the metastasis of renal cell cancer cells by inducing autophagy via AMPK/mTOR signaling pathway. Cancer Sci..

[50] Yin C (2019). G9a promotes cell proliferation and suppresses autophagy in gastric cancer by directly activating mTOR. FASEB J..

[51] Luan W (2019). Akt/mTOR-mediated autophagy confers resistance to BET inhibitor JQ1 in ovarian cancer. OncoTargets Ther..

[52] Wang LL, Zhang L, Cui XF (2019). viaDownregulation of long noncoding RNA LINC01419 inhibits cell migration, invasion, and tumor growth and promotes autophagy inactivation of the PI3K/Akt1/mTOR pathway in gastric cancer. Therap. Adv. Med. Oncol..

