# Supplementary material for: VEGF knockdown enhances radiosensitivity of nasopharyngeal carcinoma by inhibiting autophagy through the activation of mTOR pathway
Source: Sci Rep. 2020 Oct 1;10:16328. doi: 10.1038/s41598-020-73310-x (PMC7531011; doi:10.1038/s41598-020-73310-x)

VEGF knockdown enhances radiosensitivity of nasopharyngeal carcinoma by inhibiting autophagy through the activation of mTOR pathway

Li Chen[1,2*], Guoxiang Lin[1*], Kaihua Chen[2], Fangzhu Wan[2], Renba Liang[2], Yongchu Sun[2], Xishan Chen[2], Xiaodong Zhu[1,2,3]

[1]Department of Oncology, Affiliated Wuming Hospital of Guangxi Medical University, Nanning, Guangxi, 530010, People's Republic of China; [2]Department of Radiation Oncology, Guangxi Medical University Cancer Hospital, Nanning, Guangxi 530021,People's Republic of China; [3]Guangxi Key Laboratory of Early Prevention and Treatment for Regional High Frequency Tumor, Guangxi Medical University, Nanning, Guangxi 530021, People's Republic of China.

Corresponding author: Xiaodong Zhu, Department of Radiation Oncology, Guangxi Medical University Cancer Hospital, 71 He Di Road, Nanning 530021, People's Republic of China. Tel: +86 771 533 1466. E-mail address:zhuxdonggxmu@126.com.

[*] These authors contributed equally to this work.

Supplementary Figure.S1

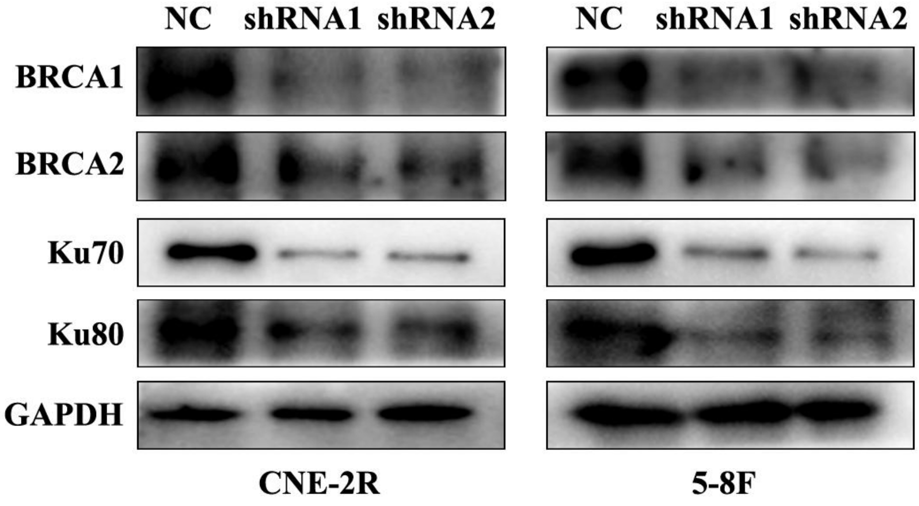

Supplementary Figure. S1. VEGF knockdown decreased the proteins of DNA repair pathways in NPC cells.

Supplementary Figure.S2

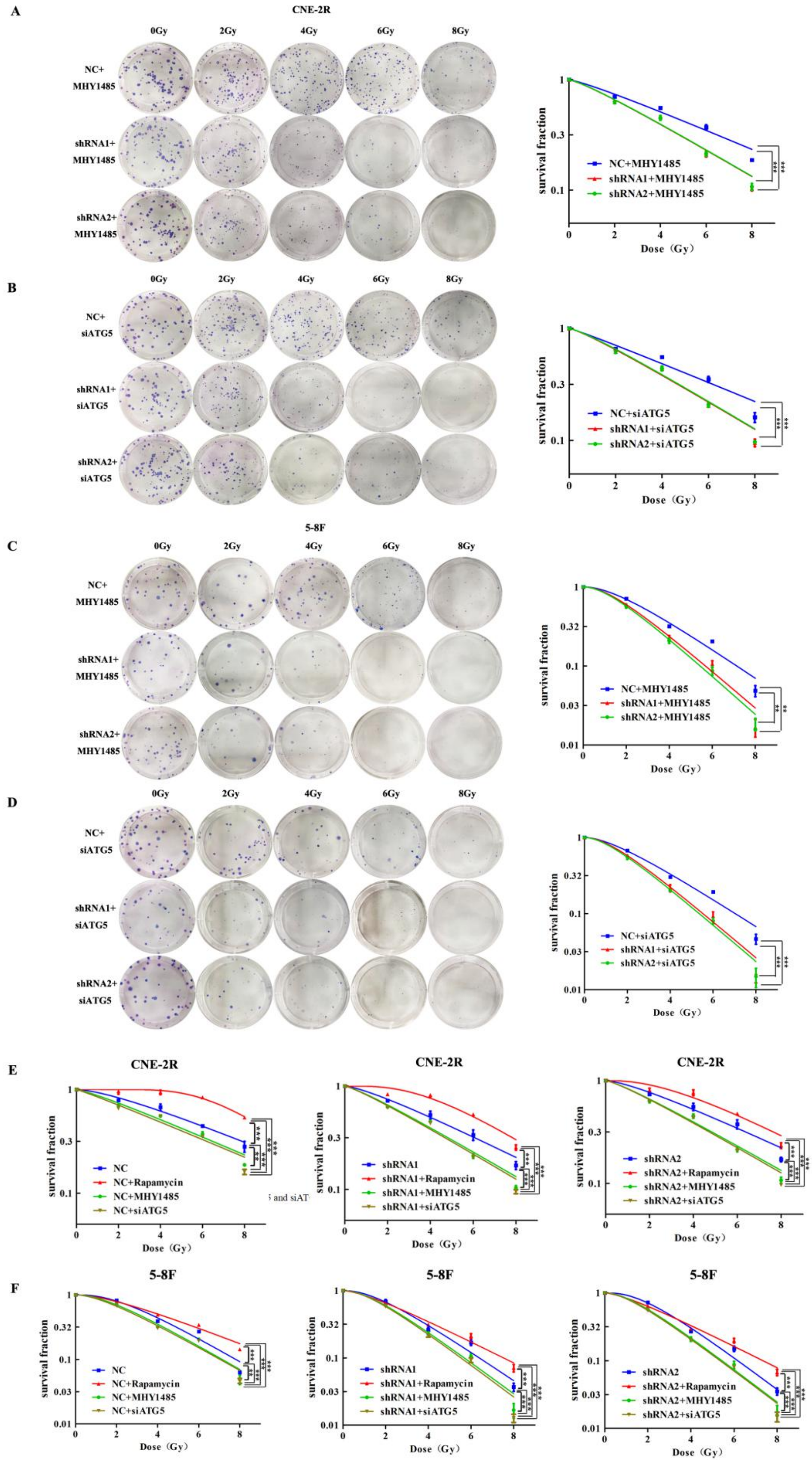

Supplementary Figure.S2 Inhibition of autophagy enhanced the radiosensitvity in NPC cells. (A-D) The number and size of colonies were analyzed by clonogenic assay after irradiation in NPC cells treated with MHY1485 and siATG5 (A, B: CNE-2R; C, D: 5-8F). (E-F) The curves showed the survival fraction of each group was detected by clonogenic assay after irradiation in NPC cells treated with or without Rapamycin, MHY1485 and siATG5 (E: CNE-2R; F: 5-8F). *$p<0.05$, **$p<0.01$ and ***$p<0.001$.

Supplementary Figure.S3

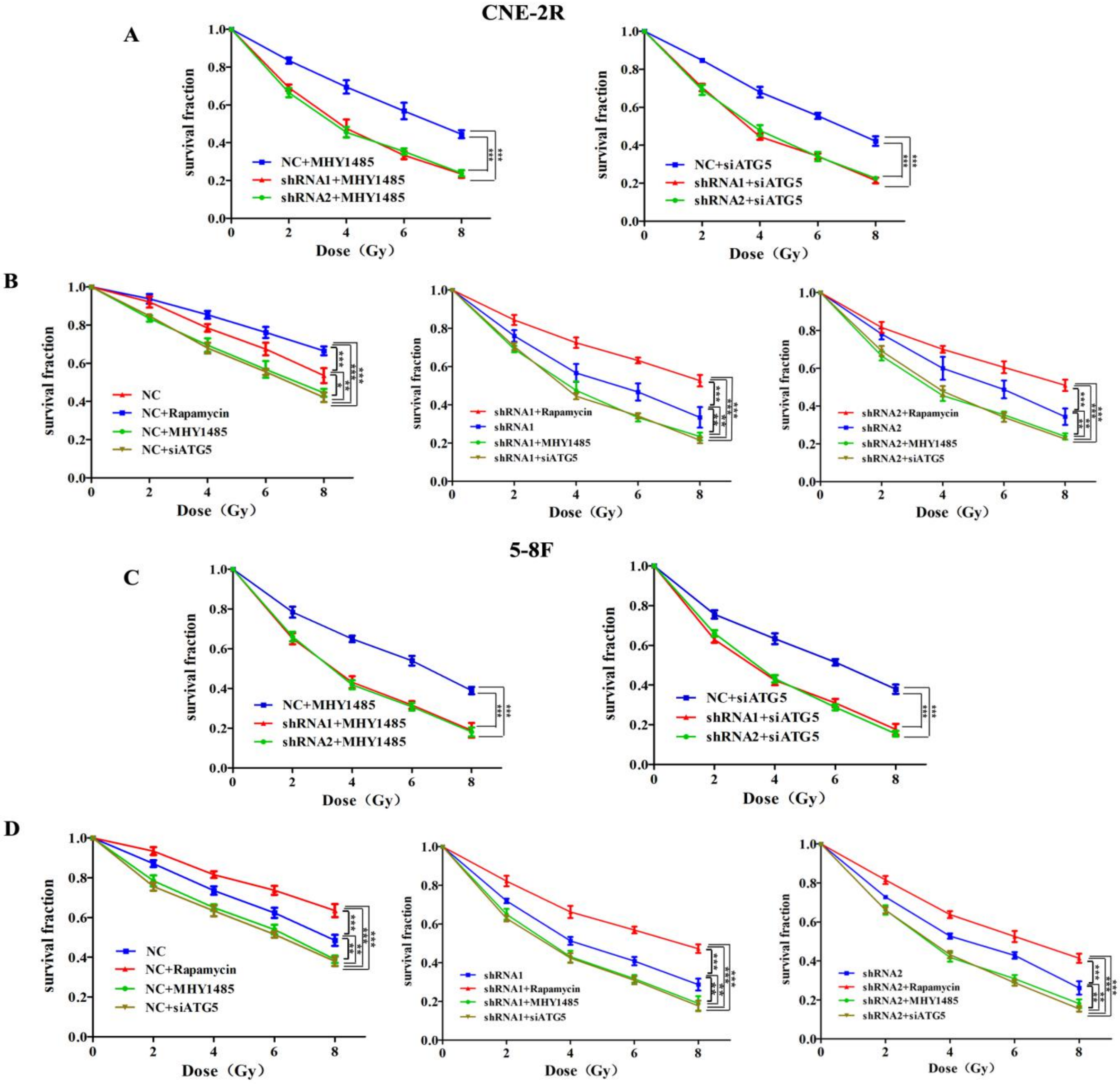

Supplementary Figure.S3 The inhibition of autophagy enhanced the radiosensitvity in NPC cells. (A,C) Cell proliferation were detected by CCK-8 assay after irradiation of NPC cells treated with MHY1485 and siATG5 (A: CNE-2R; C: 5-8F). (B,D) The curves showed the survival fraction of each group was detected by CCK-8 assay after irradiation in NPC cells treated with or without Rapamycin, MHY1485 and siATG5 (B: CNE-2R; D: 5-8F). *p<0.05, **p<0.01 and ***p<0.001.

Supplementary Figure.S4

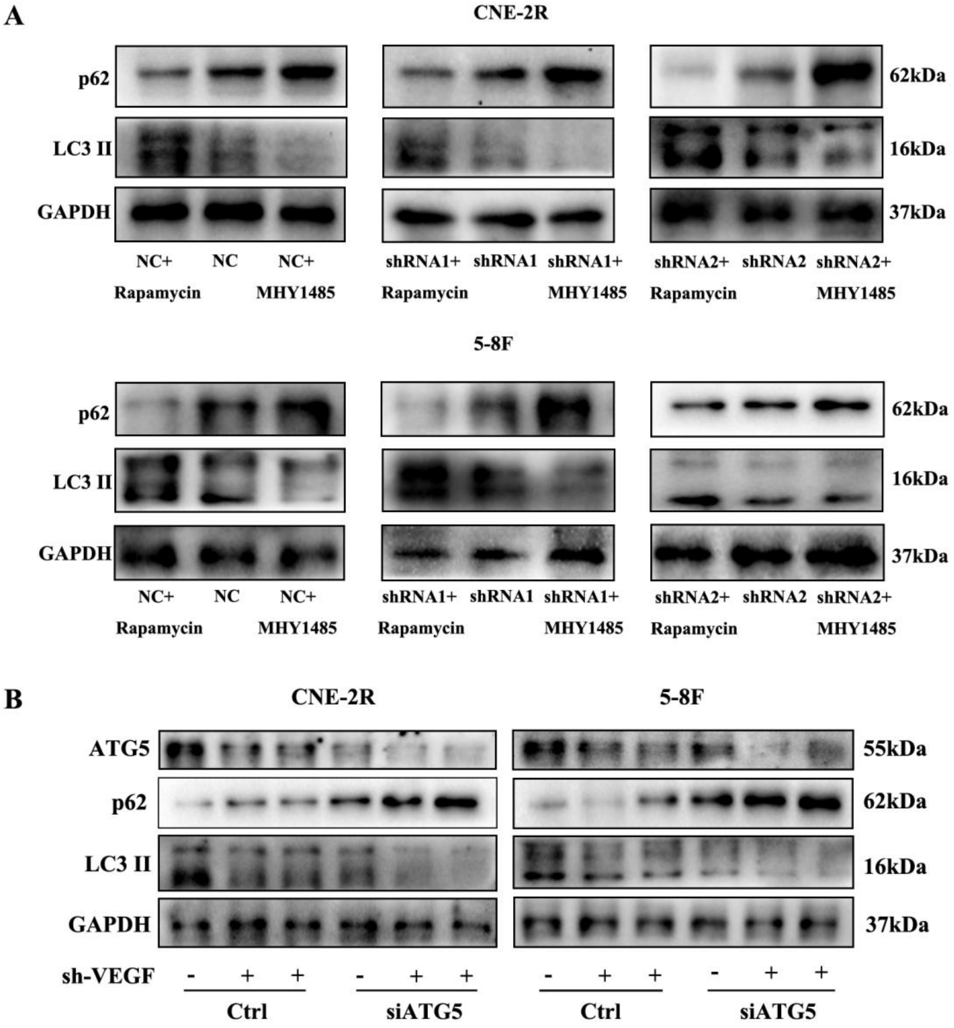

Supplementary Figure.S4 VEGF knockdown inhibited autophagy in NPC cells. (A,B) The expression of LC3 II and p62 proteins in NPC cells after treatment with Rapamycin and MHY1485. (C) The expression of LC3 II, p62 and atg5 proteins in NPC cells after treatment with siATG5.

Supplementary Table S1. Radiobiological parameters of the three groups (Mean±SD)

Cell lines (CNE-2R)	D_0	Dq	SF2
NC+Rapamycin	2.836±0.871	7.257±0.189	0.964±0.063
ShRNA1+Rapamycin	3.084±0.366	4.685±0.343	0.833±0.023
ShRNA2+Rapamycin	3.717±0.681	3.729±0.836	0.807±0.036
p	0.324	0.000	0.01

D_0 is the average lethal dose, which is the dose required to hit each cell once. Dq is a quasi-threshold dose, which refers to the ability to repair sublethal injury. SF_2 is the survival fraction at 2 Gy radiation dose. $P<0.05$ is supposed to indicate statistical significance.

Supplementary Table S2. Radiobiological parameters of the three groups (Mean±SD)

Cell lines (5-8F)	D_0	Dq	SF_2
NC+Rapamycin	2.945±0.216	2.865±0.085	0.866±0.019
ShRNA1+Rapamycin	2.46±0.187	2.225±0.202	0.776±0.03
ShRNA2+Rapamycin	2.468±0.083	2.048±0.158	0.748±0.024
p	0.021	0.002	0.003

D_0 is the average lethal dose, which is the dose required to hit each cell once. Dq is a quasi-threshold dose, which refers to the ability to repair sublethal injury. SF_2 is the survival fraction at 2 Gy radiation dose. $P<0.05$ is supposed to indicate statistical significance.

Supplementary Table S3. Radiobiological parameters of the three groups (Mean±SD)

Cell lines (CNE-2R)	D_0	Dq	SF_2
NC+MHY1485	4.882±0.047	1.023±0.204	0.704±0.007
ShRNA1+MHY1485	3.569±0.066	0.848±0.176	0.628±0.014
ShRNA2+MHY1485	3.580±0.353	0.856±0.390	0.633±0.025
p	0	0.685	0.003

D_0 is the average lethal dose, which is the dose required to hit each cell once. Dq is a quasi-threshold dose, which refers to the ability to repair sublethal injury. SF_2 is the survival fraction at 2 Gy radiation dose. $P<0.05$ is supposed to indicate statistical significance.

Supplementary Table S4. Radiobiological parameters of the three groups (Mean±SD)

Cell lines (CNE-2R)	D_0	Dq	SF_2
NC+siATG5	5.054±0.341	0.455±0.393	0.657±0.025
ShRNA1+siATG5	3.507±0.049	0.767±0.059	0.618±0.007

ShRNA2+siATG5	3.496±0.310	0.828±0.449	0.626±0.025
p	0.001	0.42	0.13

D_0 is the average lethal dose, which is the dose required to hit each cell once. Dq is a quasi-threshold dose, which refers to the ability to repair sublethal injury. SF_2 is the survival fraction at 2 Gy radiation dose. $P<0.05$ is supposed to indicate statistical significance.

Supplementary Table S5. Radiobiological parameters of the three groups (Mean±SD)

Cell lines (5-8F)	D_0	Dq	SF_2
NC+MHY1485	4.882±0.047	1.023±0.204	0.704±0.007
ShRNA1+MHY1485	3.569±0.066	0.848±0.176	0.628±0.014
ShRNA2+MHY1485	3.580±0.353	0.856±0.390	0.633±0.025
p	0	0.685	0.003

D_0 is the average lethal dose, which is the dose required to hit each cell once. Dq is a quasi-threshold dose, which refers to the ability to repair sublethal injury. SF_2 is the survival fraction at 2 Gy radiation dose. $P<0.05$ is supposed to indicate statistical significance.

Supplementary Table S6. Radiobiological parameters of the three groups (Mean±SD)

Cell lines (5-8F)	D_0	Dq	SF_2
NC+siATG5	2.405±0.108	1.556±0.114	0.673±0.015
ShRNA1+siATG5	1.821±0.111	1.380±0.132	0.581±0.021
ShRNA2+siATG5	1.801±0.081	1.250±0.226	0.553±0.036
p	0.001	0.151	0.003

D_0 is the average lethal dose, which is the dose required to hit each cell once. Dq is a quasi-threshold dose, which refers to the ability to repair sublethal injury. SF_2 is the survival fraction at 2 Gy radiation dose. $P<0.05$ is supposed to indicate statistical significance.

Original western blots images

Figure.1A

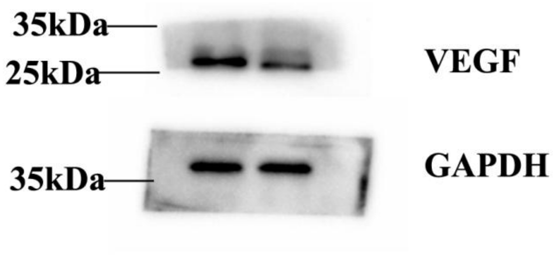

Figure.1B

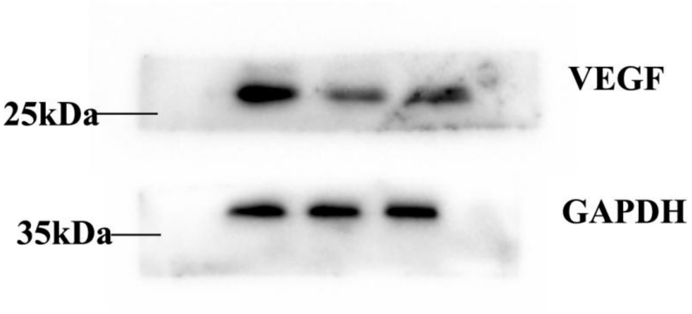

Figure.1C

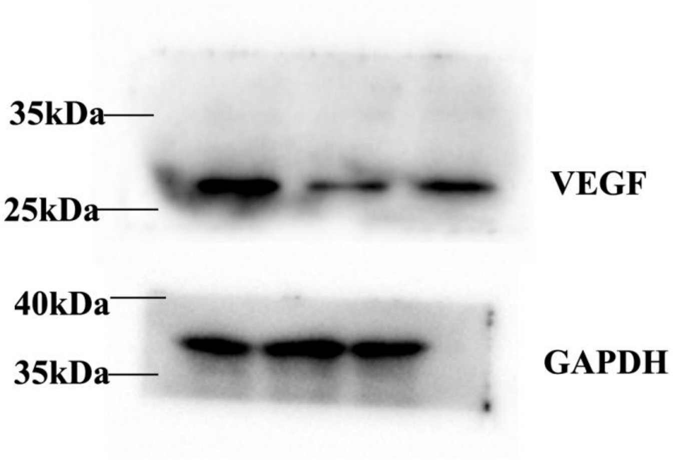

Figure.3C

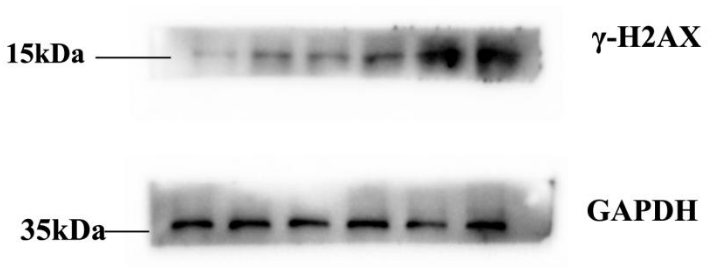

Figure.4A

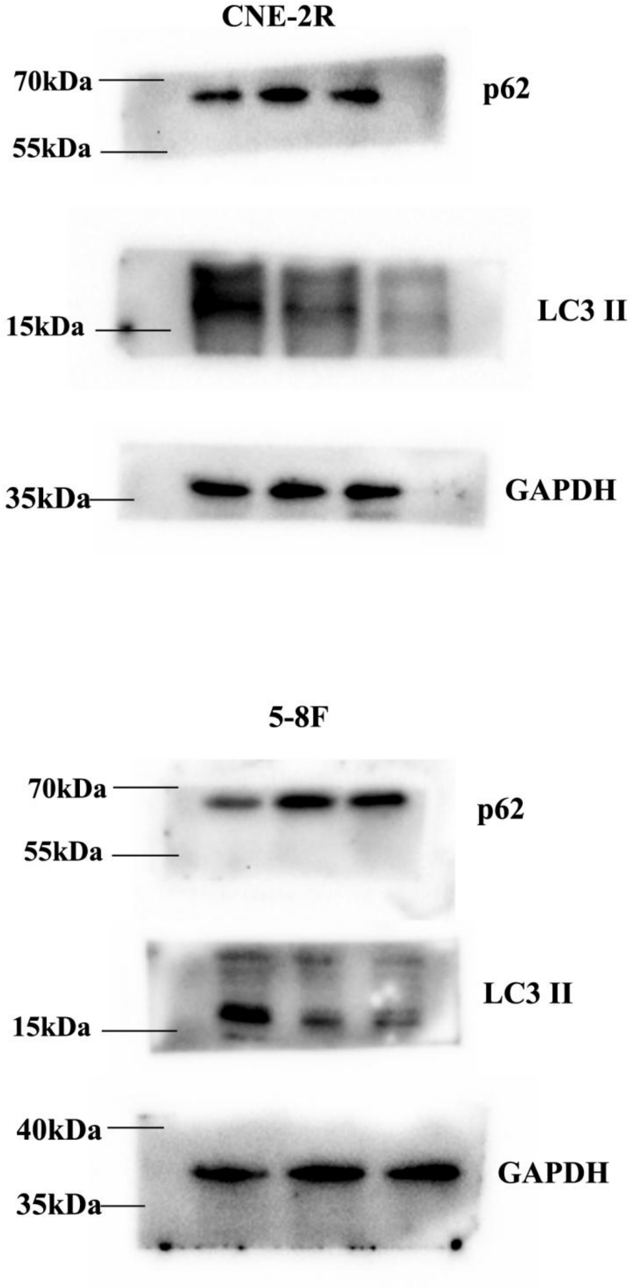

Figure.5A

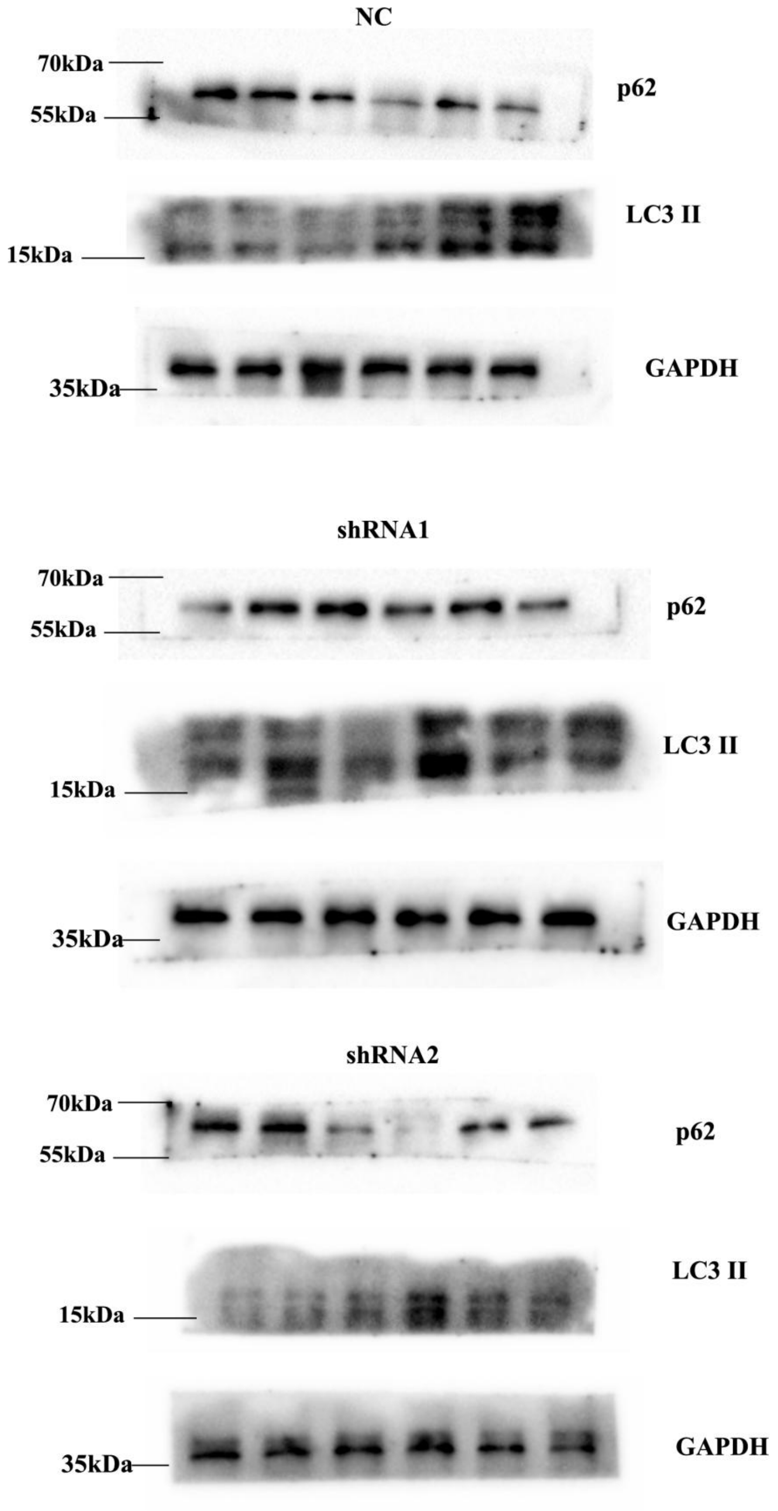
NC
70kDa
55kDa
p62
LC3 II
15kDa
GAPDH
35kDa
shRNA1
70kDa
55kDa
p62
LC3 II
15kDa
GAPDH
35kDa
shRNA2
70kDa
55kDa
p62
LC3 II
15kDa
GAPDH
35kDa

Figure. 6A

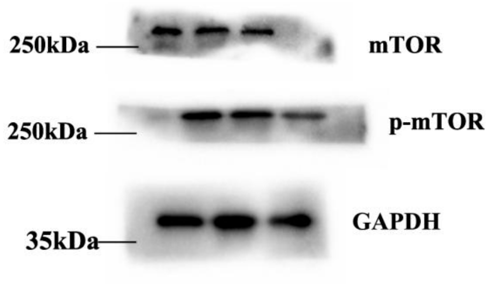

Figure. 6B

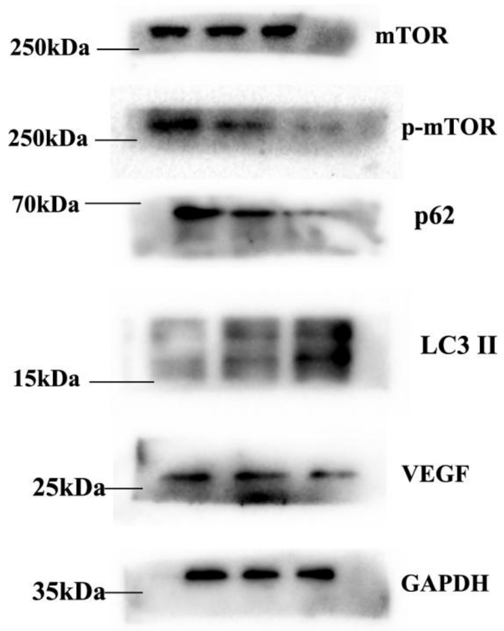

Figure. 6C

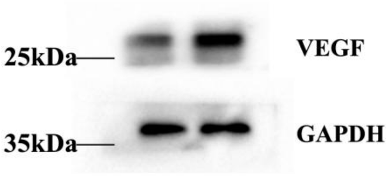

Figure. 6E

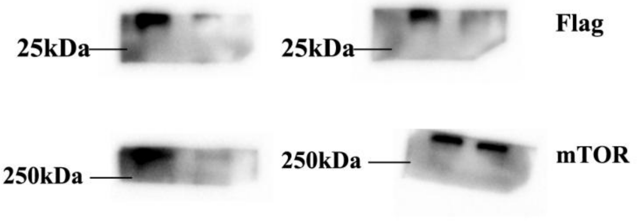

Figure. S1

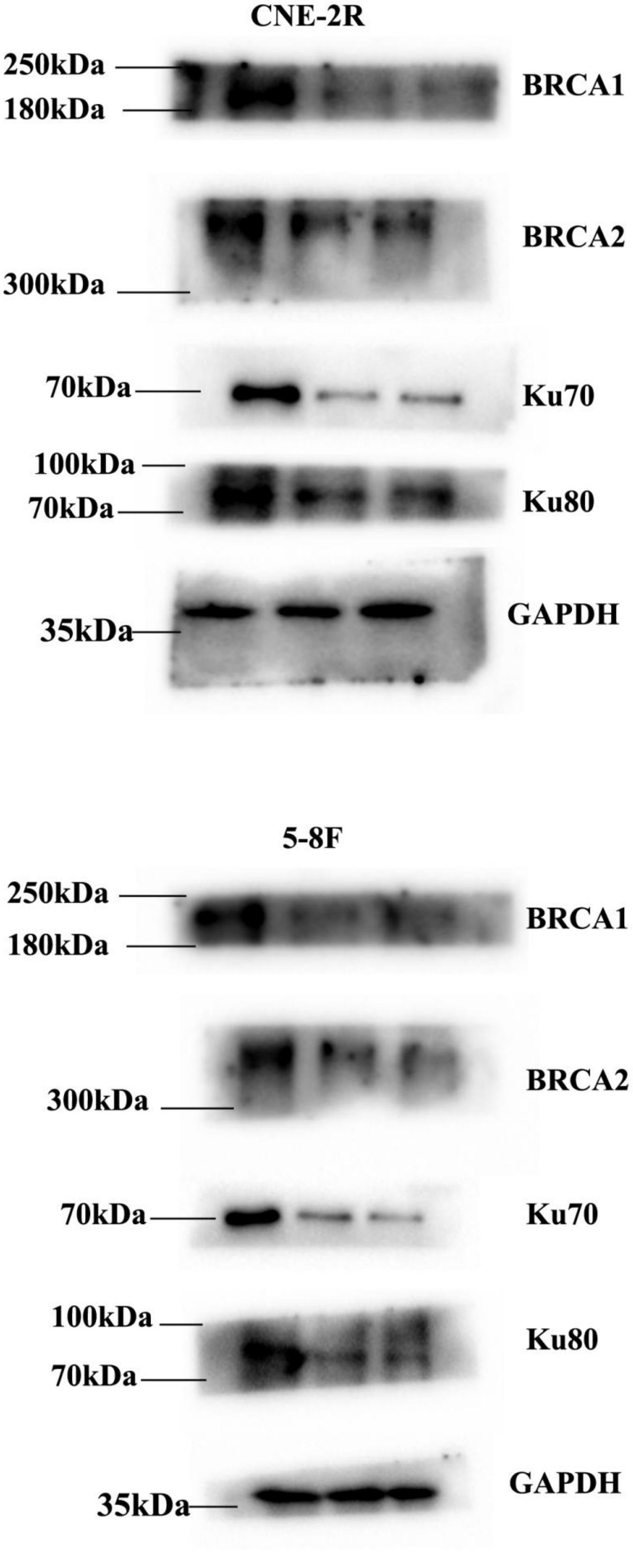
CNE-2R
250kDa
180kDa
BRCA1
BRCA2
300kDa
70kDa
Ku70
100kDa
70kDa
Ku80
GAPDH
35kDa
5-8F
250kDa
180kDa
BRCA1
BRCA2
300kDa
70kDa
Ku70
100kDa
70kDa
Ku80
GAPDH
35kDa

Figure. S4A

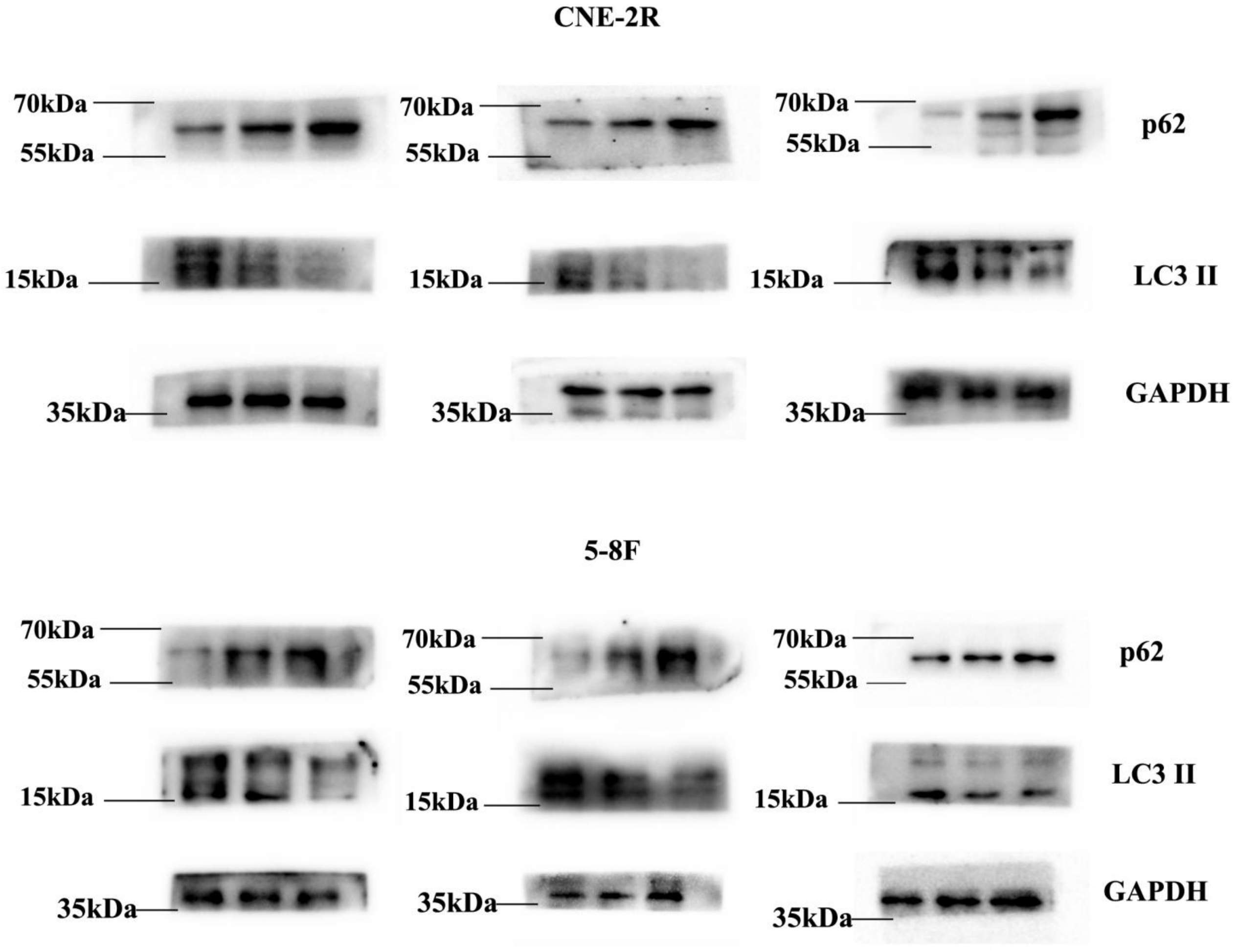

Figure. S4B

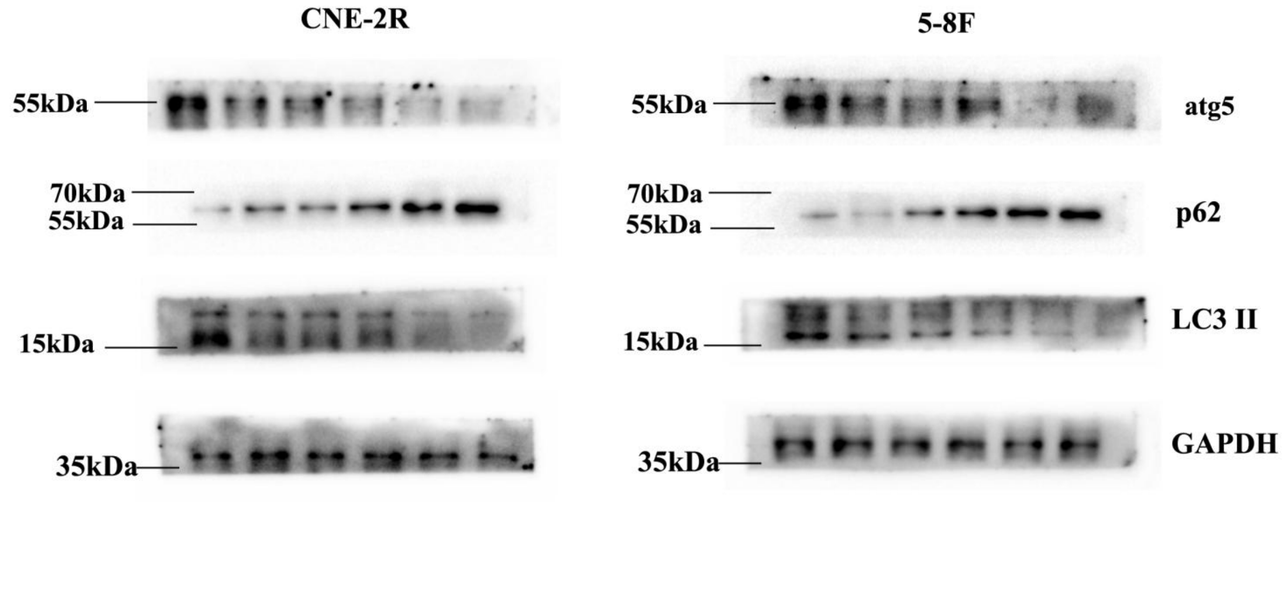

Supplement: Supplementary file 1 — Supplementary information. [file 41598_2020_73310_MOESM1_ESM.pdf]
